# On the use and misuse of externalist approaches in psychiatry

**DOI:** 10.3389/fpsyg.2013.00785

**Published:** 2013-11-13

**Authors:** Gerhard C. Bukow

**Affiliations:** ^1^Institute of Philosophy, University of MagdeburgMagdeburg, Germany; ^2^Institute of Psychology, University of GiessenGiessen, Germany; ^3^Fachbereich 06 - Psychologie und Sportwissenschaft, Abteilung Allgemeine Psychologie und Kognitionsforschung, Justus-Liebig-Universität GießenGiessen, Germany

**Keywords:** externalism, extended cognition, reliability, biological psychiatry, philosophy of science

Walter (2013) argues in that a bunch of externalist approaches examined in the theory of mind is interesting for psychiatry—especially for biological psychiatry. The externalist approaches state that mental states are not only constituted by internal but also by external factors. He subsumes them under the so-called 4E-thesis: embodied, embedded, extended, enacted. His example is concerned with ADHD such that ADHD is only existent “in an environment that favors attentional distraction and punishes hyperactivity.”

First, using the 4E-thesis seems to be explanatorily attractive: There are factors of the disorder that can be grouped as external constituents and these constituents are not named by internalist vocabulary. For example, punishment is for sure not part of internalist vocabulary.

But there is a figure of a principal argument against using the 4E-thesis in psychiatry. Consider this cookbook theory of the 4E-thesis:

Cookbook theory for the 4E-thesis in accord with Henrik Walter's multilevel-approach:

1. There is the phenomenon of a psychiatric disorder instanced in the form of multilevel-complexes.
2. There are *embodied*, *embedded*, *extended*, or *enacted* (4E) constituents of cognition that realize the cognitive systems.
3. There are individuation criteria for the 4E-constituents.
4. Coupled systems of individuated 4E-constituents individuate a multilevel-complex.
5. There are individuation criteria for systems of constituents C that realize multilevel-complexes.

For example, with respect to extended cognition, the cookbook theory is the following:

Cookbook theory of extended cognition:

1. There is the phenomenon of cognition of a group of patients in psychiatry instanced in the form of cognitive systems of a specific structure.
2. There are vehicles of cognition that realize the cognitive systems named in 1.
3. There are individuation criteria for vehicles named in 2.
4. Coupled systems of individuated vehicles individuate a cognitive system.
5. There are individuation criteria for systems of vehicles of cognition that realize cognitive systems named in 1.

As one can see, every attempt of the 4E-thesis needs criteria marking where to *stop* adding externalist constituents to the constituent base of the psychiatric disorder. For example, extended cognition-constituents should not include the whole world to make the thesis an interesting thesis—otherwise every phenomenon to be explained by extended cognition is constituted by the same constituents (i.e., the whole world). But what kind of criterion can the 4E-externalist provide us with?

First, the 4E-externalist could give a criterion based on intrinsic properties of systems realizing disorders. But as a detailed analysis of criteria of this kind by Adams and Aizawa (2001, 2010) shows, these criteria are regularly based on finding a mark of the cognitive—which seems to be a hopeless endeavor without being a fundamentalist who just adds *axiomatically* this criterion to the cookbook theory.

But second, the 4E-externalist could maybe give a criterion based on extrinsic properties of systems realizing psychiatric disorders. For example, one could use *reliability* as a stop-criterion for delineating what is coupled (with respect to point 4 in the cookbook theories). What is *reliably coupled* builds a constituent base of a system realizing a psychiatric disorder. But as a detailed analysis shows, this is not the case (Bukow and Will, in press). With respect to different criteria of reliability, there are very different reliable systems. This is not trivial, because in accord with the cookbook theory-approach, Bukow and Will argue:

Argument for the arbitrariness of the reliability-predicate:

1. Assume the cookbook theory of extended cognition.
2. Give a definition of reliability with an operationalization (e.g., reproducibility as an operationalization).
3. Choose an experimental procedure to test for reliability based on interaction between systems (because reliability is relative to this experimental procedure).
4. Choose a value X%, i.e., that the experimental procedure is reproducible in X% of all series of experiments.

There is *no* established decision procedure for the needed choices in points 3 and 4. And whatever procedure you choose, you may get different values for X% which will lead the psychiatrist to different constituent bases of the psychiatric disorder to be explained. Bukow and Will analyze in detail other candidates for criteria based on extrinsic properties, for example functional roles, causal roles, or heuristics.

Now, there are two horns for the 4E-externalist:

Horn 1: Use a stop-criterion based on intrinsic properties. The critics of Adam and Aizawa bite the proponent of this kind of criterion. If you add the criterion axiomatically, then you are a fundamentalist and not a scientist.

Horn 2: Use a stop-criterion based on extrinsic properties. Then, there is the need to show in detail how to use such a criterion—which seems to be hopeless in accord with the investigation above. If you add the criterion without detailed analysis, then you are using *just so-stories*, without guarantee that the criterion fulfills its job.

For these reasons, using the 4E-thesis is tempting but dangerous—there is no well-founded *stop-criterion* in philosophy of mind that a psychiatrist could use—not in a detailed way and not in a rough way. It is arbitrary what type and what token the psychiatrist may use with respect to the selection of constituents of a disorder. In a principal debate, the 4E-proponent may excuse these deficits because nobody may give in principal such stop-criteria. But in psychiatry, principal deficits are no excuse for misuse. Arbitrariness can smooth the way for use—but also for misuse in psychiatry, which is a dangerous zone with respect to rights and needs of patients of psychiatry. And if a psychiatrist can only give arbitrary constituent bases to a patient's disorder, then disorder and treatment will only arbitrarily be defined. This is, as far as I can see, an objection against Henrik Walter's argument for a mature “third wave” biological psychiatry.
